# Non-Hebbian Learning Implementation in Light-Controlled Resistive Memory Devices

**DOI:** 10.1371/journal.pone.0052042

**Published:** 2012-12-14

**Authors:** Mariana Ungureanu, Pablo Stoliar, Roger Llopis, Fèlix Casanova, Luis E. Hueso

**Affiliations:** 1 CIC nanoGUNE Consolider, Donostia - San Sebastian, Spain; 2 LPS, CNRS - UPS, Bât. 510, Orsay, France; 3 ECyT, UNSAM, San Martín, Buenos Aires, Argentina; 4 IKERBASQUE, Basque Foundation for Science, Bilbao, Spain; Florida International University, United States of America

## Abstract

Non-Hebbian learning is often encountered in different bio-organisms. In these processes, the strength of a synapse connecting two neurons is controlled not only by the signals exchanged between the neurons, but also by an additional factor external to the synaptic structure. Here we show the implementation of non-Hebbian learning in a single solid-state resistive memory device. The output of our device is controlled not only by the applied voltages, but also by the illumination conditions under which it operates. We demonstrate that our metal/oxide/semiconductor device learns more efficiently at higher applied voltages but also when light, an external parameter, is present during the information writing steps. Conversely, memory erasing is more efficiently at higher applied voltages and in the dark. Translating neuronal activity into simple solid-state devices could provide a deeper understanding of complex brain processes and give insight into non-binary computing possibilities.

## Introduction

Ramón y Cajal postulated that the nervous system is formed of individual fundamental units called neurons linked to each other by small contacts [1], that were later called synapses [2]. In spite of the astonishing continuous progress made in neuroscience for more than a century, there is still a lack of understanding of many neuronal mechanisms, mainly due to their complexity and versatility. For example, for the specific case of neuronal processes, Hebb proposed that its basis stands on the synaptic strength (weight) increase caused by the simultaneous activity of both presynaptic and postsynaptic neurons [3]. The learning process proposed by Hebb is inherently unstable because of the so-called autocorrelation aspect. In simple terms, autocorrelation represents the trend of the synaptic weight for self-amplification, that is, the more a synapse drives a postsynaptic cell the more the synaptic weight will grow. Likewise, once depressed, the synaptic weight decreases invariably to zero. One realistic way of stabilizing the synaptic weight is to introduce an extra, third factor capable of modulating the learning process so as to control the self-amplification. Such third factors are typically neuromodulators and are usually inputs external to the analyzed synaptic system. An example of an external neuromodulator is dopamine in certain brain areas manifested by mechanisms of learning and forgetting processes, for example in classical or operant conditioning [4]–[7]. In addition to the experimental influence of diverse neuromodulators, mathematical models in neuronal networks have demonstrated their role in the plasticity of memory processes [8]–[9].

Resistive memories are solid-state devices in which a resistance state can be set by an appropriate sequence of voltage pulses of well-determined durations [10]–[14]. This behavior resembles some key aspects of synapses in the brain, since the voltage pulses act very much like the neuronal action potentials (or spikes) in Hebbian processes [15], [16]. In biological synapses the learning process is strengthened by its repetition, and a similar behavior has also been observed in solid-state resistive memory devices mimicking Hebbian learning [17]–[21].

In this paper we move a step further and present the first experimental implementation of a three-factor non-Hebbian learning in a single memory device. In this specific example, we employ a light-controlled resistive memory device [22]. In our system the electrical resistance (which is equivalent to the synaptic weight) is modulated not only by the voltage (equivalent to neuronal action potentials) as in conventional resistive memories, but also by a third, external factor, which in our case is the presence of light. Moving closer to translating complex neuronal operations into simple solid-state devices can provide both a deeper understanding of neuromodulated brain processes and give insight into non-binary computing possibilities.

## Results and Discussion

**Figure 1:**
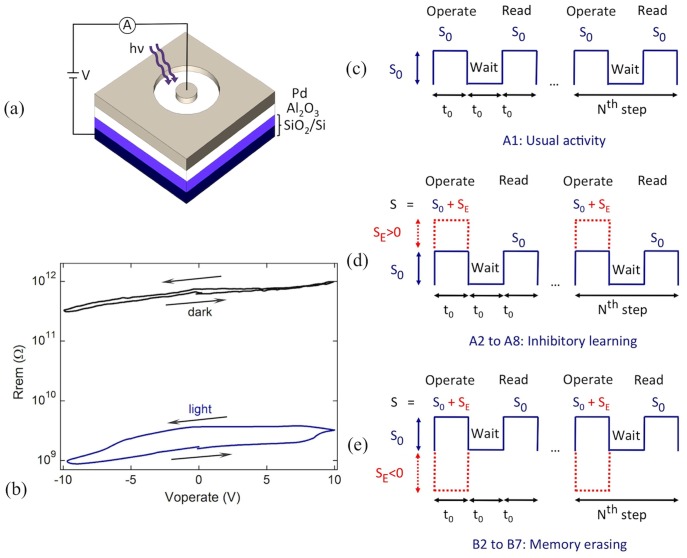
Device and measurements protocols. (a) Configuration of the light-controlled resistive memory devices. 20 nm of Al_2_O_3_ are deposited on p-Si/SiO_2_. The bottom electrode is the Si substrate, whereas the top electrode is Pd patterned by photolithography. Light can reach the optically active Si substrate through the spaces uncovered by Pd and after crossing the transparent Al_2_O_3_ and SiO_2_ layers. Inside the Si, under illumination, charge carriers are photogenerated. (b) Typical remnant resistance hysteresis switching loops measured at 7 V after applying a voltage pulse between -Voperate and Voperate either in the dark or under illumination, followed by a 100 ms waiting time at 0 V to discharge capacitive effects. (c-e) Steps of voltage-pulses applied to the memory in different learning processes (summarized in Table 1 and 2); the sequence is for example repeated 1000 times in the case of the ‘usual activity’ measurements (process A1 in Table 1). S_0_ is the typical voltage input and is applied in the usual activity or in the reading steps, while S_E_ is the extra-external voltage input, applied in addition to S_0_ during the inhibitory learning (S_E_>0) or the Memory erasing (S_E_<0 and |S_E_|>|S_0_|) steps. Processes A2–A6 (Table 1) contain ‘inhibitory learning’ steps (figure d), meaning that positive S voltages are applied a number of times. Processes B1–B4 (Table 2) comprise ‘Memory erasing’ steps (figure e), meaning that negative S voltages are applied a number of times. The operating voltage pulse (S_0_ or S = S_0_+S_E_) is applied either in the dark or with light, as a third extra-control parameter, followed by a waiting time in short-circuit conditions (at 0 V) and then followed by the measurement of the induced memory state by a reading voltage of S_0_ = 7 V under illumination. The step time t_0_ is 100 ms in all cases.

In Figure 1a we show the design of a light-controlled resistive memory. It consists of a 20-nm-thick Al_2_O_3_ film deposited on a p-doped Si substrate covered with a thin layer (1.9 nm) of native SiO_2_. The Si substrate works as bottom electrode in a metal-oxide-semiconductor (MIS) configuration while Pd electrodes, patterned by photolithography, act as top electrodes. Light can reach the optically active silicon substrate through the spaces uncovered by palladium and after crossing the transparent aluminum oxide and silicon oxide layers. The behavior of the light-controlled memory devices is based on the photogeneration of charge carriers in silicon under illumination. With suitable applied voltage-pulses, the photogenerated electrons from Si are injected in the Al_2_O_3_ layer. A fraction of these electrons is then trapped in the Al_2_O_3_ layer, changing quasi-permanently its resistance state [22]. The electrical characterization of the devices was performed by means of remnant resistance hysteresis switching loops (HSL; Figure 1b). Each step on the HSL represents the remnant resistance (Rrem) measured at 7 V either in the dark (curve labeled ‘dark’ in Figure 1b) or under illumination (the curve labeled ‘light’ in Figure 1b), after sweeping the voltage between −Voperate and +Voperate (each pulse of the sweep has a length of 100 ms), again either with light or in the dark, respectively. Each Voperate pulse is followed by a waiting time of 100 ms at 0 V to discard capacitive effects before finally reading the device state with the 7 V voltage pulse. In the dark, the absence of photogenerated electrons in Si results in a non-hysteretic remnant resistance HSL curve. Under illumination, the resistance decreases strongly due to the presence of photogenerated charge carriers in the system, and a hysteretic non-volatile memory behavior is obtained. The HSL proves that a non-volatile memory state can be intrinsically defined by a chosen voltage pulse under illumination, although it also depends on any previous memory state.

**Table 1:** Summary of the inhibitory learning processes tested.

Process	‘Operate’ voltage pulses				
	*Usual activity, S_0_*	Inhibitory learning,S = S_0_+S_E_ (S_E_>0)	*Usual activity, S_0_*	Inhibitory learning,S = S_0_+S_E_ (S_E_>0)	*Usual activity, S_0_*
A1	*1000x (S_0_, light)*				
A2	*100x (S_0_, light)*	500x (S = 10 V, light)	*400x (S_0_, light)*		
A3	*100x (S_0_, light)*	500x (S = 10 V, dark)	*400x (S_0_, light)*		
A4	*100x (S_0_, light)*	500x (S = 8 V, light)	*400x (S_0_, light)*		
A5	*100x (S_0_, light)*	500x (S = 8 V, dark)	*400x (S_0_, light)*		
A6	*100x (S_0_, light)*	500x (S = 10 V, light)	*100x (S_0_, light)*	50x (S = 10 V, light)	*250x (S_0_, light)*

**Figure 2:**
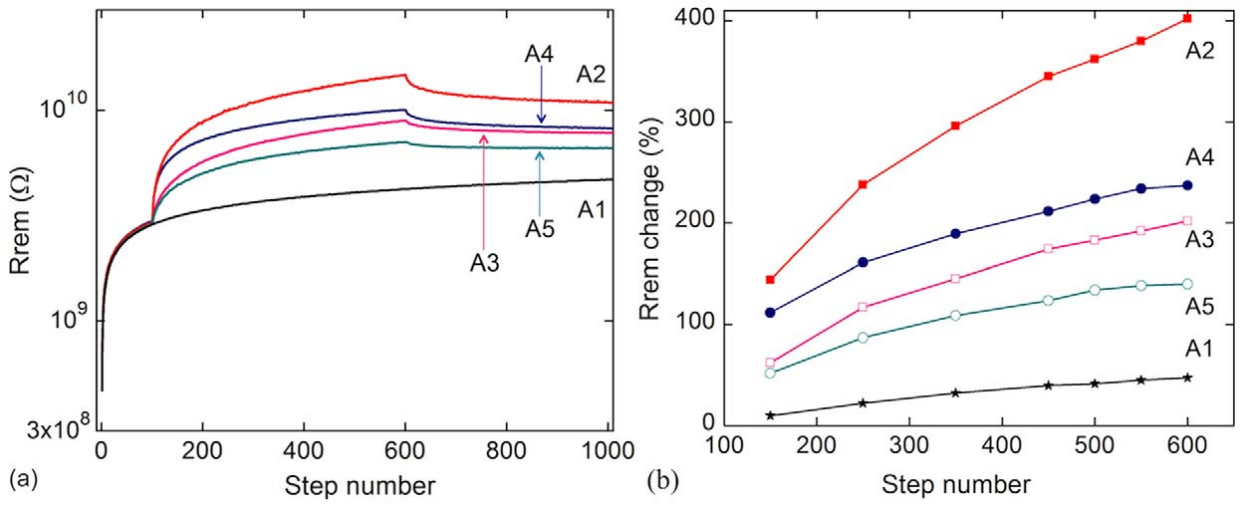
Inhibitory learning of light controlled resistive memory devices. (a) Output of the different processes used for analyzing the efficiency of the different ‘inhibitory learning’ processes. The remnant resistance was measured at S_0_ = 7 V with light after the inhibitory learning voltage pulses (S_0_+S_E_) were applied either in the dark or with light. The curves are labeled following the protocols described in Table 1. (b) Efficiency of the different learning processes described in Table 1. Processes A2, A4 are inhibitory learning with light, whereas processes A3, A5 are learning in the dark. Approximately double efficiency is obtained when light is present during learning as compared to learning in the dark. A1 is the ‘usual activity’ curve, which serves as a base line.

**Figure 3:**
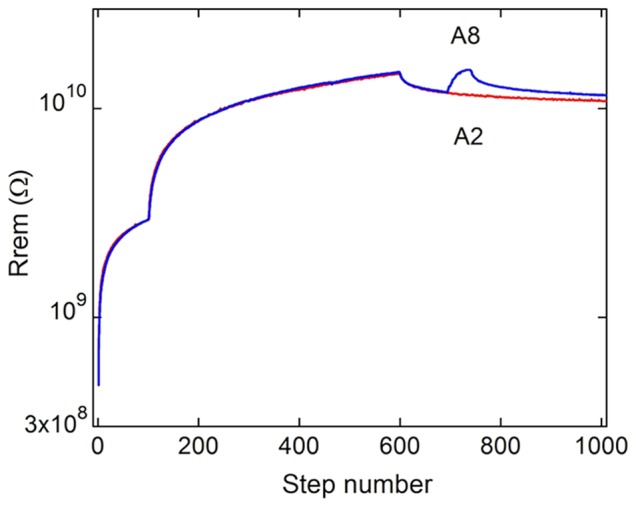
Output remnant resistance measured in processes A2 and A6, as described in **Table 1**. After a learning process followed by a forgetting time, the system only needs a small reminder to reach back to the ‘well-learned’ state. This behavior is similar to processes encountered in living organisms.

The outcome of the different processes is displayed in Figures 2 and 3. For processes A2–A6 the first 100 voltage pulses sets are the same, followed by different inhibitory learning V-sets. S_0_ = 7 V in all cases and represents ‘usual activity’.

After this brief initial presentation and characterization of basic properties of the light-controlled resistive switching devices, we move now to the testing of different learning processes, thus showing the influence of the external non-Hebbian third factor in the results. In Figures 1c to 1e we present the individual different voltage-pulses steps that constitute the core of the different processes implemented. We have denoted them as ‘Usual activity’ (Fig. 1c), ‘Inhibitory learning’ (Fig. 1d) and ‘Memory erasing’ (Fig. 1e). The measuring sequence for each point of these processes is: I. the application of a 100 ms Operate voltage pulse (of different amplitudes, S_0_ or S, in dark or under illumination), II. the application a 100 ms waiting step in short-circuit conditions for discharging any capacitive effects, and finally III. the measurement of the state of the memory system under illumination (please refer to the Experimental Section for details) with a voltage pulse S_0_ = 7 V of 100 ms duration. For segment I. of the measurement sequence presented above, the Operate voltage pulse is applied in the dark for processes A3, A5 (only during the Inhibitory learning part, as presented in Table 1) and B2, B4 (only during the Memory erasing part, see Table 2) and under illumination for all other processes. In all the different protocols tested we keep the same conditions for reading the remnant resistance (Rrem) state of the memory device, namely S_0_ = 7 V under illumination, in order to sense the changes created by the different learning activities performed in the ‘Operate’ step. These different core steps are repeated a certain number of times during the learning processes presented in this paper (summarized in Tables 1 and 2). Before the beginning of each process (presented in Tables 1 and 2) a cleaning protocol is performed with the aim of removing any trapped charges in the Al_2_O_3_ layer. This cleaning consists in 400 pulses of −10 V, 100 ms each, in the dark, and we observed that this protocol is equivalent to bringing the memory to a pristine state, as all past events are fully erased.

**Table 2:** Summary of the different ‘inhibitory learning’ and ‘Memory erasing’ processes tested.

Process	‘Operate’ voltage pulses			
	*Usual activity, S_0_*	Inhibitory learning,S = S_0_+S_E_ (S_E_>0)	Memory erasing,S = S_0_+S_E_ (S_E_<0)	*Usual activity, S_0_*
B1	*100x (S_0_, light)*	500x (S = 10 V, light)	100x (S = −10 V, light)	*300x (S_0_, light)*
B2	*100x (S_0_, light)*	500x (S = 10 V, light)	100x (S = −10 V, dark)	*300x (S_0_, light)*
B3	*100x (S_0_, light)*	500x (S = 10 V, light)	100x (S = −7 V, light)	*300x (S_0_, light)*
B4	*100x (S_0_, light)*	500x (S = 10 V, light)	100x (S = −7 V, dark)	*300x (S_0_, light)*

**Figure 4:**
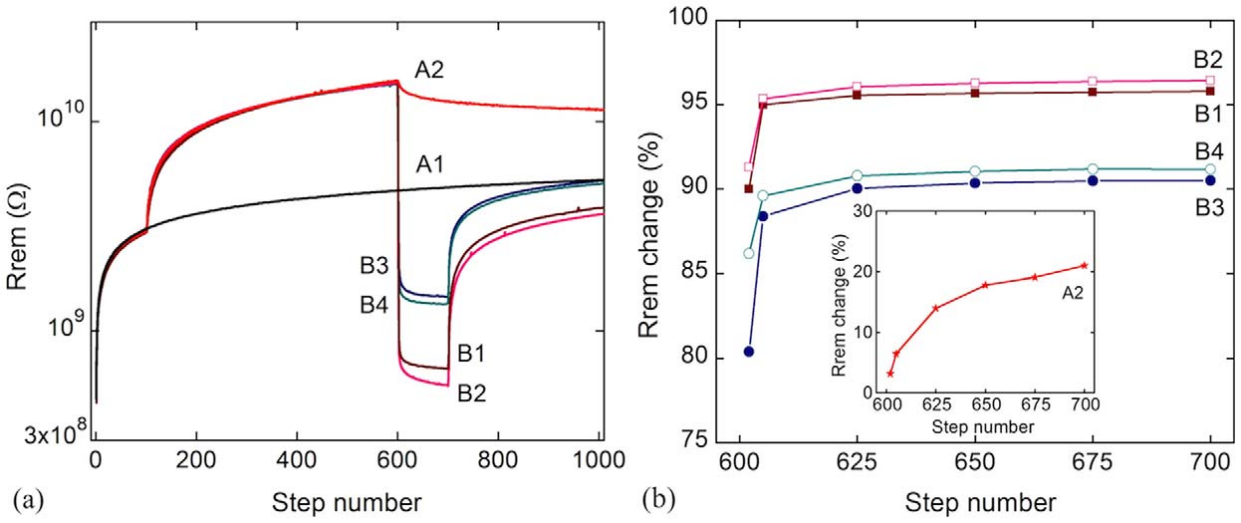
Output of the different processes used to analyze the efficiency of memory erasing through positive reinforcement voltage pulses. The remnant resistance is read at S_0_ = 7 V under illumination. Process A1 represents usual activity (see Table 1). A2 is a process without any erasing step and information is slowly forgotten over time (starting from step 600). Processes B1, B3 contain negative-voltages Memory erasing steps performed under illumination, while processes B2, B4 contain Memory erasing steps in the dark. Additionally, a more efficient memory erasing is obtained with higher-amplitude negative voltages and in the dark.

For processes B1 to B4 the first 600 sets of voltage pulses are the same, followed by different memory erasing voltage pulses. S_0_ = 7 V in all cases. The outcomes are presented in Figure 4.

For positive applied voltages S>0, the remnant resistance increases (see Figure 1b), while at negative applied voltages S<0, the remnant resistance decreases. Within this paper we identify the former step as an inhibitory learning process, while we associate the latter step with erasing the learned information. The inhibitory learning in our device correlates with a neuronal plasticity-rule where the synaptic strength is reduced through the learning process. In this context, the opposite process, excitatory learning, would be a learning process involving the increase of synaptic strength (i.e. decrease of resistance).^18^ The step we refer to as erasing step resets the synaptic weight to a reproducible initial condition with low resistance.

In Table 1 we summarize the different ‘inhibitory learning’ processes tested (the outcomes of the processes are presented in Figures 2 and 3). Process A1 (Figure 2a) is equivalent to the experience achieved during a usual day-to-day activity (the applied voltage pulses are shown in Figure 1c) as we observe that the system learns slowly over time (the resistance increases monotonically). We can further study the inhibitory learning by applying different sets of pulses, distinct from process A1 (the usual activity). Process A2 departs from A1 after the application of the 100 initial voltage pulses sets. In this particular case we continue by applying 500 voltage pulses of S = 10 V under illumination (in the ‘Operate’ step, see Figure 1d), followed by 400 steps of S_0_ = 7 V that represent again a usual activity protocol (Figure 1c). Other protocols follow similar routines although we change the amount and amplitude of the inhibitory learning part of the protocol.

In Figure 2 we show the behavior of our system after different learning sequences (A1 to A5, Table 1). As described above, the process A1 is equivalent to the experience achieved during a usual day-to-day activity, the system learns slowly over time. Distinctly, processes A2–A5 contain steps that are equivalent to applying inhibitory stimuli on a bio-system (S_E_>0). The learning is faster than in case A1, and this is represented by a more pronounced permanent increase of the remnant resistance of the device (in agreement with Figure 1b, with light). A stronger stimulus or a higher number of events lead to a more efficient inhibitory learning, as it also happens in Hebbian learning (Figure 2b). In addition, the presence of light as an extra, external (to the two interconnected neurons) control parameter during learning increases its efficiency.

After the inhibitory learning steps (N >600 in processes A2–A5), the system returns to the usual activity protocol and starts forgetting the information. That is, learning is only maintained if the stimulus is present and forgetting occurs as an exponential decay.

The key point of our work is that in addition to the normal inhibitory learning, we obtain higher learning efficiency when the system is illuminated. This external light input is the equivalent of a third factor that controls learning in bio-organisms. This is more evident in Figure 2b, where we summarize the efficiency of the different learning processes. We define the efficiency of each inhibitory learning process as the change in the electrical resistance induced in the inhibitory learning part of the overall protocol and in the following form: abs(‘Rrem at step 100’ - ‘Rrem after inhibitory learning pulses’)×100/‘Rrem at step 100’. From Figure 2b it is clear that the remnant resistance change (that is, the efficiency of the inhibitory learning process) is higher in the processes performed under illumination (A2, A4) as compared to the corresponding equivalent processes in the dark (A3, A5). It is especially noticeable that the light irradiation approximately doubles the efficiency of the inhibitory learning process as compared to similar processes performed in the dark (see Figure 2b).

In Figure 3 we present the comparison between protocols A2 and A6. Protocol A6 is equivalent to protocol A2 for the 700 initial sets of V-pulses, after which we have introduced a second inhibitory learning step (50 pulses, 10 V, with light) and a final ‘usual activity’ routine. The comparison between both protocols presents another example of similitude between the learning processes in bio-systems and in light-controlled resistive memories. After a process in which information was efficiently learned (in our case in steps 200 to 600), followed by a time of normal forgetting, the system only needs a small reminder to reach back to the ‘well-learned’ state.

In Table 2 we summarize the different processes in which we investigate the efficiency of erasing the information previously learned by the system (Figure 4 shows the outcome of these processes). The first 600 voltage-pulses sets in processes B1 to B4 are the same as in processes A2 to A6, a usual activity part followed by an inhibitory learning part. These 600 pulses are followed by different information-erasing voltage sets. Forgetting of inhibitory-information in bio-systems is accelerated by positive reinforcements, in our case by applying negative voltage pulses to the device (S<0, voltage pulses presented in Figure 1e).

Figure 4a shows the behavior of the memory system after the different Memory erasing processes. The last 400 steps in process A2 (Table 1) are equivalent to a slow, continuous, day-to-day forgetting in bio-systems and are here exemplified by a progressive decrease in the electrical resistance after the inhibitory learning factors have been removed. Processes B1 to B4 (in which we also include erasing steps with negative ‘Operate’ voltage pulses; see Table 2) are the equivalent of applying a positive-reinforcement on a bio-system after the inhibitory learning took place. Information is forgotten more efficiently in the dark and with higher-amplitude stimuli, meaning higher-amplitude negative applied voltage pulses (−10V, processes B1, B2, Rrem is more sharply decreasing). This effect can be observed comparing processes B1, B2 at −10 V and B3, B4 at −7 V (in agreement with Figure 1b). Note that most of the learned information is forgotten within the first two steps of the applied erasing voltages, nevertheless a higher number of erasing pulses lead to better memory cleaning (Figure 4b). The Rrem change in this case is defined as: abs((‘Rrem at step 600’ - ‘Rrem after Memory erasing pulses’) *100/‘Rrem at step 600’). As it can be observed, information can be efficiently erased with an adequate set of voltage pulses. Again in this case, the processes studied (Table 2, information removal) have a different efficiency in the dark or under illumination. Dark conditions lead to a slightly higher efficiency (B2, B4) than under illumination (B1, B3), highlighting again the role of the external third factor in the memory process.

## Methods

The 20-nm-thick Al_2_O_3_ films were prepared on p-Si/SiO_2_ by atomic layer deposition at 300°C from trimethylaluminium (TMA) as metal-carrying precursor gas and H_2_O vapour as oxygen source, separated by N_2_ inert gas purges. We then covered the entire surface of the Al_2_O_3_ with Pd except the rings left around the circular top contacts for the light to enter through the transparent oxide layers and reach the optically active Si substrate. The radius of the circular top Pd contacts is 50 µm. The radius of the uncovered oxide rings around the top metal contacts is 1 cm for the results presented in this paper. Equivalent results can be obtained for ring-radii in the range 1 mm to 1 cm, since at about 1 mm we reach the electron diffusion length in the lightly doped p-Si substrates [22].

For measurements under illumination, the samples were irradiated with 2.5 mW/cm^2^ UV light using a light emitting diode (LED) situated at 3 cm distance from sample and having a wavelength of 390 nm.

The reading of the remnant resistance, Rrem, was always performed under light, only the writing/erasing conditions were different, either under light or in the dark. We chose to read the remnant resistance with light in all measurements (2.5 mW/cm^2^ UV) in order to better verify the system state, because when reading R_rem_ in the dark we always obtain a value in the 10^11^ Ω range. This large resistance in the dark is due to the limited amount of free-electrons present in the system, since there is no photogeneration of charge carriers in the Si substrate (the main source of free electrons in our system).

### Conclusions

We have analyzed the behavior of light-controlled resistive memory devices [22] as synaptic-mimics for inhibitory learning processes. We have demonstrated that the learning processes are not only controlled by the voltage pulses applied to the device (that are equivalent to the neuronal action potentials) but also by an external third factor, in our case the presence of light. This is, to the best of our knowledge, the first implementation of a non-Hebbian learning process in a single solid-state device. We believe that light-controlled non-volatile resistive memory devices offer a new perspective for the investigation of neuromodulated learning processes. More complex structures, bringing together a number of devices that would act as neuronal networks, could be the next step into implementation of biological processes into silicon-compatible architectures.
